# Unique Clone of *Coxiella burnetii* Causing Severe Q Fever, French Guiana

**DOI:** 10.3201/eid1907.130044

**Published:** 2013-07

**Authors:** Aba Mahamat, Sophie Edouard, Magalie Demar, Philippe Abboud, Jean-Yves Patrice, Bernard La Scola, Antoine Okandze, Félix Djossou, Didier Raoult

**Affiliations:** Centre Hospitalier Andree Rosemon, Cayenne, French Guiana (A. Mahamat, M. Demar, P. Abboud, A. Okandze, F. Djossou);; Université des Antilles-Guyane, Cayenne (A. Mahamat, M. Demar, P. Abboud, F. Djossou);; Aix Marseille Université, Marseille, France (S. Edouard, J.-Y. Patrice, B. La Scola, D. Raoult)

**Keywords:** *Coxiella burnetii*, bacteria, clone, Q fever, genotyping, multispacer sequence typing, Cayenne, French Guiana

## Abstract

Acute Q fever is an emergent and severe disease in French Guiana. We obtained 5 *Coxiella burnetii* isolates from samples of patients from Cayenne and found an epidemic clone circulating in Cayenne. This clone has caused pneumonia and endocarditis and seems to be more virulent than previously described strains.

Q fever, which is caused by the bacterium *Coxiella burnetii*, has rapidly emerged in French Guiana since 1996 (*1*). The incidence of acute Q fever in the capital, Cayenne, is one of the highest in the world. The annual incidence of Q fever was estimated at 37 cases/100,000 persons in 1996 (*2*) and increased to 150 cases/100,000 persons in 2005 (*3*). The most common clinical feature of Q fever in Cayenne is pneumonia, and *C. burnetii* is the causative agent of 24% of all community-acquired pneumonias (*4*). These forms of acute Q fever are particularly severe (*4*). Subsequently, we have hypothesized the existence of a specific source of *C. burnetii* responsible for human infections, which is unidentified to date, and the existence of a different strain of *C. burnetii* that circulates in Cayenne.

Q fever is diagnosed by serologic analysis in Cayenne. *C. burnetii* is rarely identified by PCR and has yet to be cultured in Cayenne. In this study, we isolated 5 *C. burnetii* strains from biologic samples of patients from Cayenne. We compared the strains from Cayenne with other strains and showed that a unique genotype is circulating in Cayenne. This unique genotype might be related to the clinical and epidemiologic features of severe fever in Cayenne.

## The Study

As a national reference center for Q fever, our center receives samples from France and other regions for serologic, molecular, histologic, and immunohistochemical analyses as described (*5**–**7*). In 2012, we received a cardiac valve sample from a patient in Cayenne with Q fever endocarditis who had undergone surgery in Martinique. In the same year, we collected 4 heparinized blood samples from patients with acute Q fever from Cayenne that were collected before initiation of antimicrobial drug treatment.

**Figure 1:**
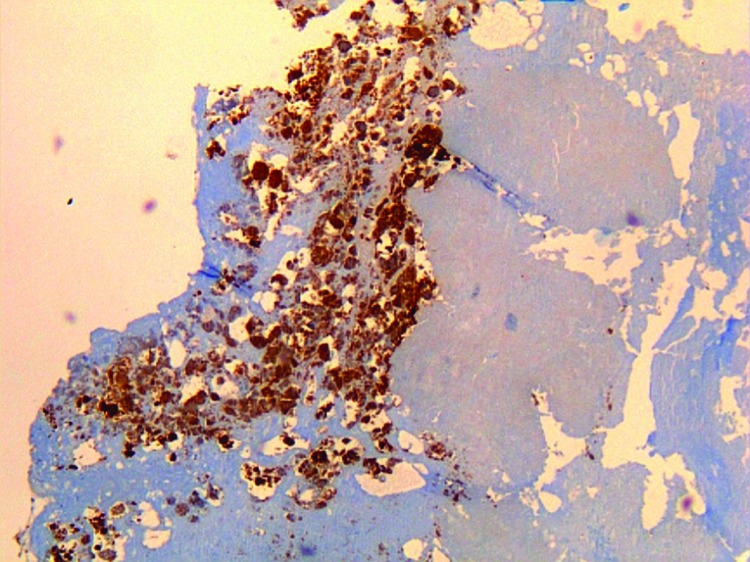
Immunohistochemical detection of *Coxiella burnetii* in resected cardiac valve of a 60-year-old man with Q fever endocarditis, Cayenne, French Guiana. Monoclonal antibody against *C. burnetii* and hematoxylin were used for staining. Original magnification ×50.

The patient in Cayenne who had endocarditis was a 60-year-old man. Serologic titers of IgG1 increased to 51,200 in this patient, and results of quantitative PCR (qPCR) and immunohistochemical analyses of the valvular sample were positive for *C. burnetii* (Figure 1). The other 4 patients (2 men and 2 women) had fever and acute pneumonia, and 2 of them also had increased transaminase levels. All 4 patients had serologic titers compatible with acute Q fever. The heparinized blood samples were tested by using a specific qPCR; samples from only 1 patient were positive for *C. burnetii*.

**Figure 2:**
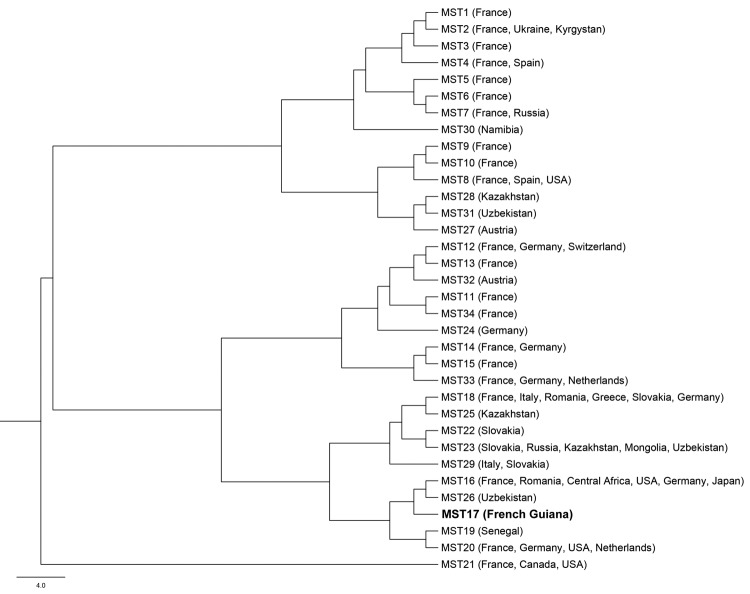
Phylogenetic diversity of 34 genotypes of *Coxiella burnetii* identified by using multispacer sequence typing (MST). Clone from French Guiana isolated in this study is indicated in **boldface**. Scale bar indicates nucleotide substitutions per site.

All samples were cultured as described (*8*). We successfully cultured *C. burnetii* from the valvular sample after incubation for 16 days and from 3 blood samples after incubation for 25, 32, and 32 days. Paradoxically, the only blood sample that was positive by qPCR was negative by culture. Genotyping was performed by using multispacer sequence typing for intergenic regions (*9*). We identified the isolate from the cardiac valve as genotype 17. Spacers Cox 51 and Cox 20 were the most discriminating spacers in identifying this genotype, and the 3 isolates from the blood cultures were also identified by these 2 spacers as genotype 17 (Figure 2). This genotype had been identified in our laboratory only once, in 2000 in an aortic valve of a 40-year-old French man who had undergone surgery in France, had Q fever endocarditis, and was co-infected with *Streptococcus oralis*. Retrospectively, we found that patient had lived in Cayenne for years before his diagnosis with Q fever endocarditis. Therefore, all genotype 17 isolates were obtained from who lived or had lived in Cayenne, making it unique to this area. We determined the antimicrobial drug susceptibilities of these isolates, and the MIC of doxycycline was 0.25 µg/mL for all isolates (*10*).

For the past 10 years, routine cell culture for *C. burnetii* has been performed in our laboratory. We found that the proportion of isolates obtained from blood samples of patients with acute Q fever was higher for patients from Cayenne than for patients from metropolitan France. We obtained 3 isolates from 5 blood samples from untreated patients in Cayenne and 3 isolates from 65 samples from patients in metropolitan France (p = 0.003, by Fisher exact test). However, we did not find any difference with respect to the culture delay between patients from the 2 locations.

## Conclusions

Our work shows that genotype 17, a unique genotype, is circulating in Cayenne. This genotype is related to genotypes that harbor the QpH1 plasmid, which causes the most severe clinical forms of acute Q fever in experimental animal models (*11**,**12*). Only bacteria from this group and bacteria containing the QpDV plasmid have been found in cases of human acute Q fever (*9**,**13*). We believe that severity of Q fever infection is related to the strain of *C. burnetii* circulating in Cayenne. Because the population in French Guiana is a large diversity of Creole, Amerindian, Maroon, Caucasian, and Asian persons (*14*), we excluded the hypothesis that genetic susceptibility of patients from Cayenne to *C. burnetii* infection might be related to severity of the disease.

We observed a higher rate of strain isolation from the blood of patients from Cayenne than from the blood of patients from metropolitan France. This difference may be related to a higher bacterial load in blood or a greater ability of the bacteria to grow on cell cultures. This second hypothesis seems more likely because even if the observed delay for the first culture was the same for genotype 17 and other genotypes, the strain from Cayenne was isolated more frequently in the cell line that we used (HEL cells) than other strains of *C. burnetii* from France. In addition, we did not find a higher number of DNA copies in the blood of patients from Cayenne, and qPCR results were not correlated with culture results.

*C. burnetii* isolates from Cayenne were susceptible to antimicrobial drugs, particularly doxycycline. These isolates cause acute pneumonia and endocarditis. Among the 34 genotypes identified in our study by multispacer sequence typing, 28 (82%) were associated with disease in humans. A larger biodiversity of strains has been observed in samples from patients in metropolitan France (*9*), where 21 genotypes circulate (Figure 2). In contrast, during the recent Q fever outbreak in the Netherlands, it appears that a single strain (genotype 33) was responsible for the epidemic (*15*). We believe that there has been an epidemic developing in Cayenne since 1996 that is caused primarily, if not solely, by a single strain that has circulated since at least 2000, whose reservoir is unknown (*1**,**2*).

In conclusion, *C. burnetii* genotype 17 is circulating in French Guiana and causing acute infections and endocarditis. This strain is epidemic and most likely causes more acute infections with exacerbated immune responses than other known genotypes of *C. burnetii*. Genotype 17 might be the most virulent genotype of *C. burnetii* described to date.
